# Managing Urethral Diverticulum During Pregnancy Utilizing Advanced Ultrasonographic Techniques: A Literature Review and Case Study

**DOI:** 10.3390/biomedicines13061432

**Published:** 2025-06-11

**Authors:** Desirèe De Vicari, Marta Barba, Alice Cola, Matteo Frigerio

**Affiliations:** Department of Gynecology, IRCCS San Gerardo dei Tintori, University of Milano-Bicocca, 20900 Monza, Italy; m.barba8792@gmail.com (M.B.); alice.cola1@gmail.com (A.C.)

**Keywords:** urethral diverticulum, pregnancy, ultrasonography

## Abstract

Urethral diverticulum (UD) during pregnancy is a rare clinical condition, with limited literature available to guide standardized management. Fewer than a dozen well-documented cases have been reported, but they reflect a wide range of clinical approaches from antenatal surgery to postpartum intervention. We report the case of a 36-year-old woman diagnosed at 34 weeks of gestation with a 5.5 cm urethral diverticulum, presenting with suprapubic pain, urinary dribbling, and green vaginal discharge. Conservative management was pursued due to obstetric concerns, including multiple uterine fibroids and risk of preterm labor. Advanced ultrasonographic techniques—biplane transvaginal imaging, transperineal ultrasound, and 3D surface rendering—enabled a detailed anatomical assessment of parameters including the lesion’s size, shape, and relationship to the urethra, without resorting to invasive diagnostics. The diverticulum was found to cause 90° urethral angulation and had a C-shaped configuration, with a volume of 11.5 cm^3^. Following antibiotic treatment, the patient’s symptoms improved, and she remained clinically stable. She was scheduled for vaginal delivery followed by postpartum diverticulectomy. This case illustrates the diagnostic value of high-resolution ultrasound in pregnancy and supports literature recommendations favoring conservative treatment and delayed surgery to reduce maternal and fetal risk. Vaginal delivery remains a viable option in select UD cases.

## 1. Introduction

Urethral diverticulum (UD) is a rare condition marked by a sac-like outpouching of the urethral mucosa, which communicates with the urethral lumen. While uncommon in the general population, it is particularly rare during pregnancy, with only a few documented cases [1,2]. The pathogenesis is often linked to chronic urinary tract infections (UTIs) and the obstruction or rupture of Skene’s glands, sometimes progressing to abscess formation [3,4].

Pregnancy introduces physiological changes—such as increased urinary frequency and bladder compression—that can obscure or mimic UD symptoms, delaying diagnosis [1,2]. The clinical presentation of UD may overlap with that of more common conditions, including UTIs, vaginal cysts, and fibroids. MRI is the diagnostic gold standard but is often avoided during pregnancy. Advanced transvaginal ultrasound, including biplanar and 3D imaging, has emerged as a safe, effective alternative, offering detailed anatomical insights [3].

Management is usually conservative during pregnancy, focusing on symptom relief and infection control, with surgical excision (diverticulectomy) postponed until the postpartum period [1,3,4,5].

Given the scarcity of cases and absence of standardized guidelines, clinical decision-making should be individualized and supported by a multidisciplinary team including obstetricians, urologists, and radiologists. Advances in ultrasonographic imaging have significantly improved our ability to identify and monitor UD during pregnancy, thereby informing management strategies and optimizing maternal and neonatal outcomes.

## 2. Case Report

A 36-year-old woman at 34 weeks of gestation (G2P1) presented to the obstetric unit with complaints of suprapubic pain, intermittent urinary dribbling, and green vaginal discharge. She had a previous vaginal delivery without complications and no other significant medical conditions. Her obstetric history was complicated by multiple uterine fibroids, which raised concerns about potential preterm labor. At the time of presentation, the patient was not in active labor, and her vital signs were stable, with a blood pressure of 110/70 mmHg and a heart rate of 85 beats per minute.

Upon examination, her abdomen was soft, and uterine contractions were not palpable. Pelvic examination revealed a tender area in the anterior vaginal wall, but no palpable masses were noted. Laboratory investigations, including a complete blood count (CBC) and C-reactive protein (CRP), were within normal limits, indicating no signs of acute infection or systemic inflammation.

Given her obstetric history and the risk of preterm labor, empirical broad-spectrum antibiotics were initiated, covering common pathogens such as Escherichia coli, Chlamydia trachomatis, and Neisseria gonorrhoeae, while awaiting further diagnostic workup. An indwelling catheter was inserted, draining approximately 150 mL of concentrated urine. Urine analysis showed no evidence of bacteriuria, suggesting that a urinary tract infection was unlikely.

The first imaging modality employed”was ’ransvaginal ultrasound (TVUS), performed using the Mindray Nuewa I9 system, which identified a hypoechogenic lesion lateral to the left urethra, measuring approximately 4 cm in size. The lesion was initially suspected to be a Skene’s gland cyst or a vaginal wall cyst. Given the need for further clarification, advanced imaging techniques were employed. A biplane transvaginal ultrasound was performed, followed by 3D ultrasound imaging, which provided detailed anatomical information.

**Figure 1:**
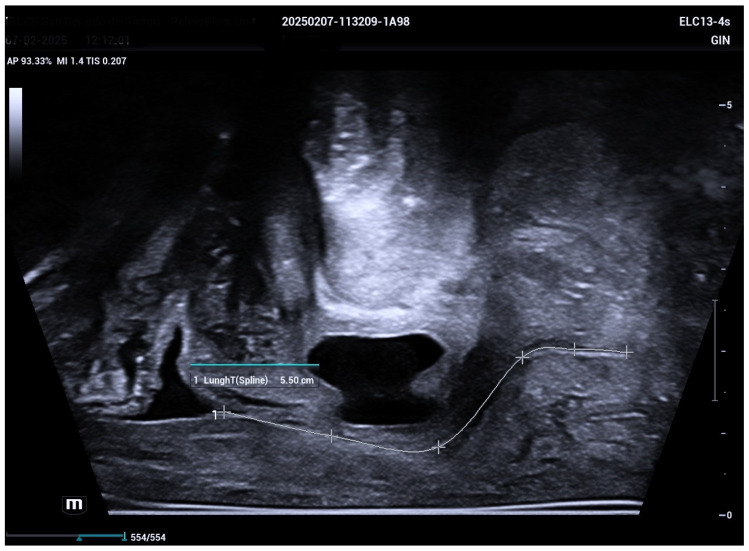
Mid-sagittal imaging using a biplanar ultrasound probe demonstrates a urethral diverticulum located approximately 1.5 cm distal to the internal urethral orifice. The diverticulum measures 3 cm in length and causes a marked 90-degree angulation of the distal urethra. The total length of the urethra is measured at 5.5 cm. This anatomical distortion may contribute to urinary symptoms and complicate vaginal delivery.

**Figure 2:**
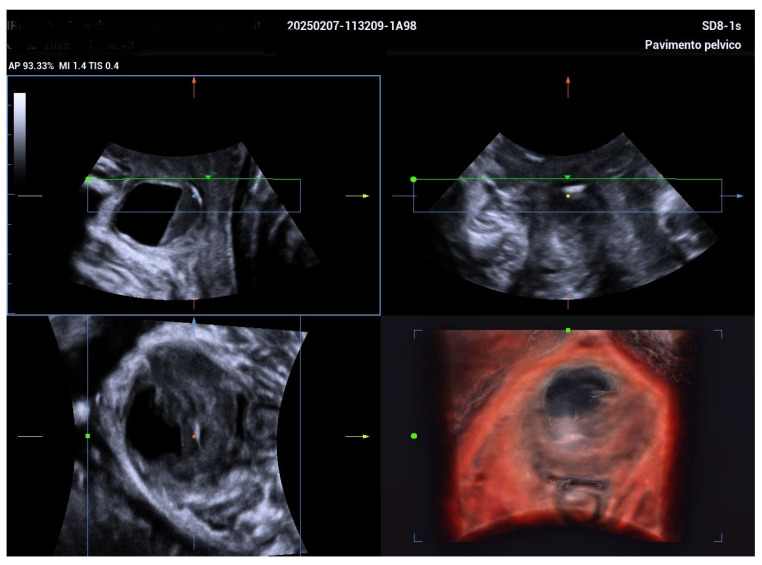
A superficial 3D rendering illustrating a C-shaped urethral diverticulum configuration encircling the urethra from the 9 o’clock to the 2 o’clock position. This shape highlights the extent and orientation of the diverticulum relative to the urethral circumference, which may influence clinical symptoms and treatment planning.

**Figure 3:**
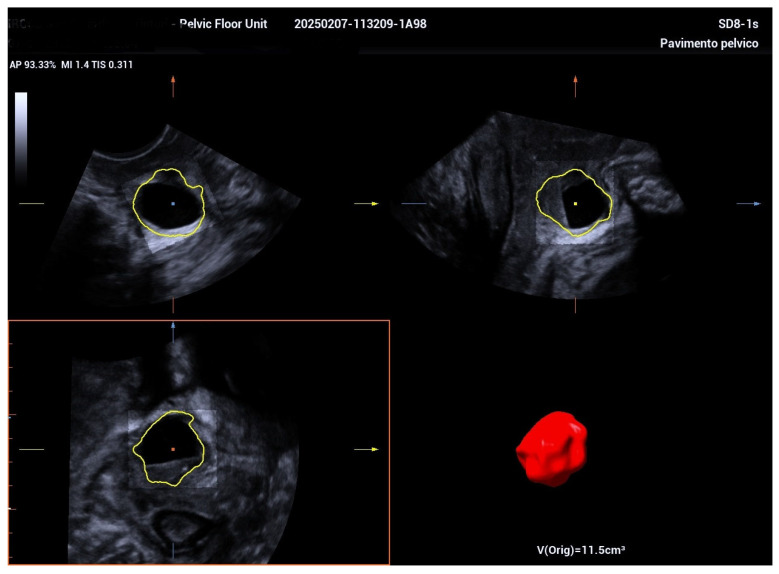
A volumetric 3D rendering of the urethral diverticulum showing a total volume of 11.5 cm^3^, providing precise quantification of its size to assist in clinical assessment and surgical planning.

**Figure 4:**
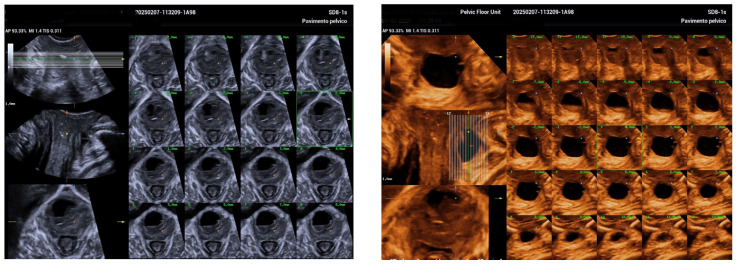
A high-resolution computed tomography (CT) scan with sequential 1 mm slices acquired in the sagittal, coronal, and transverse planes. This detailed imaging enabled precise visualization of the diverticular collar’s course and location. On the right, a 3D reconstruction further illustrates the anatomical relationships and morphology of the diverticulum.

**Figure 5:**
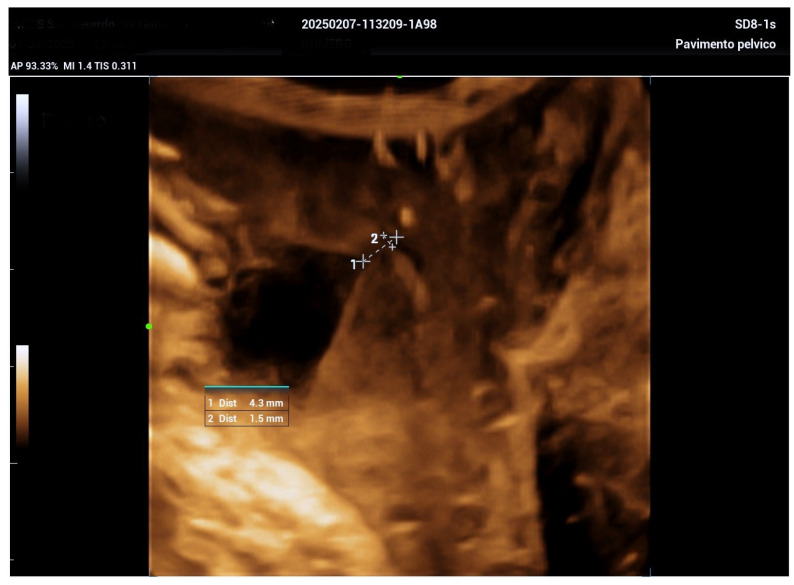
Three-dimensional evaluation of the diverticular collar, illustrating the measurement and calculation of its length and diameter to assess the precise anatomy and size of the urethral diverticulum.

The 3D ultrasound revealed a 5.5 cm diverticulum located 1.5 cm from the internal urethral orifice, with a C-shaped configuration (Figure 1). The diverticulum was oriented at a 90° angle to the urethra, with a volume of 11.5 cm^3^, and extended from the 9 o’clock to the 2 o’clock position (Figure 2 and Figure 3). The diverticular collar was identified at the 2 o’clock position, measuring 4.3 mm in length and 1.5 mm in diameter (Figure 4 and Figure 5). All procedures were conducted following institutional ethical standards, with approval from the San Gerardo Hospital Institutional Review Board (GSM-LASER 2023), ensuring patient safety and adherence to clinical protocols throughout diagnosis and management.

These findings were consistent with a diagnosis of urethral diverticulum. There was no evidence of associated urethral obstruction or other complications, such as bladder stones or malignancies.

The patient’s acute symptoms, including the urinary dribbling and green vaginal discharge, were treated with a course of antibiotics. The symptoms gradually resolved, and there was no evidence of labor progression during her hospital stay. After discussing the treatment options with the patient, it was decided to continue with conservative management, as her condition was stable and there was no indication for urgent surgical intervention. She was counseled on the risks and benefits of both vaginal delivery and cesarean section, with a focus on the potential impact of the diverticulum on the delivery process.

The patient opted for vaginal delivery, with a planned postpartum diverticulectomy to remove the diverticulum and address any potential long-term complications. This management approach was consistent with the recommendations in the literature, which suggest that conservative management is often appropriate for most pregnant women with UD, with surgery deferred until after delivery to reduce the risks associated with anesthesia and surgical intervention during pregnancy.

## 3. Discussion

Urethral diverticulum (UD) is a rare and frequently underdiagnosed condition, particularly in pregnant women, where physiological and anatomical changes in gestation can obscure typical symptoms and complicate timely diagnosis [1,5,6]. The case presented is notable for the late gestational diagnosis at 34 weeks, the diverticulum’s considerable size and anatomical impact, and the successful application of advanced three-dimensional (3D) ultrasonographic techniques to guide conservative management.

This case underscores the diagnostic challenges associated with UD during pregnancy, as its symptoms—such as urinary frequency, suprapubic pain, and incontinence—often overlap with common pregnancy-related conditions and may be misattributed to gestational urinary changes [1,3,4]. The differential diagnosis was extensive, including Gartner duct cysts, Skene′s gland cysts, vaginal inclusion cysts, and ectopic ureteroceles. These alternatives were systematically excluded through detailed clinical evaluation and high-resolution biplanar and 3D transvaginal and transperineal ultrasound, which precisely delineated the diverticular anatomy and its collar [3,6,7].

Given the absence of acute complications, surgical intervention was deferred to the postpartum period to minimize maternal and fetal risks associated with anesthesia and preterm labor [4,8]. This case illustrates the clinical value of advanced ultrasonography in achieving a safe, non-invasive diagnosis and facilitating informed, staged management.

## 4. Literature Review

The extreme rarity of urethral diverticulum (UD) during pregnancy poses significant challenges in establishing standardized diagnostic and therapeutic protocols. The absence of large-scale studies or trials forces clinicians to rely on case reports, small series, and expert opinion. The literature confirms that UD in pregnancy is an uncommon condition, with only limited cases reported over decades, hindering the development of evidence-based management guidelines.

Management is usually conservative during pregnancy, with surgery often postponed until postpartum. Conservative treatments include antibiotics, catheterization, or minimally invasive drainage for symptom relief. When surgery is necessary due to complications or failed conservative care, diverticulectomy is typically performed postpartum with favorable outcomes [3,7,8,9].

UD may lead to complications like recurrent UTIs, labor obstruction, and postpartum bladder dysfunction. Enlarged diverticula or those near the bladder neck can complicate delivery, but most patients still achieve successful vaginal births. Cesarean sections are generally reserved for unrelated obstetric reasons [5,8,10,11].

Advanced imaging plays a critical role in diagnosis and management. Transvaginal and 3D ultrasound provide high-resolution views of diverticular anatomy, aiding treatment decisions and delivery planning [3,5,7,8]. MRI, used when ultrasound is inconclusive, offers superior soft tissue detail without radiation, helping tailor management and anticipate complications [10]. Due to limited data and a lack of guidelines, UD in pregnancy requires individualized care. However, evolving imaging modalities and increasing case documentation are improving diagnosis and guiding better clinical decisions.

A systematic review of the literature was conducted in accordance with the Preferred Reporting Items for Systematic Reviews and Meta-Analyses (PRISMA) guidelines. The literature search was performed using EndNote X8 (Clarivate Analytics) to manage references and screen the results. A comprehensive search strategy was applied across multiple databases, including PubMed, Scopus, Web of Science, and the Cochrane Library, utilizing the keywords “urethral diverticulum” and “pregnancy”.

The search encompassed publications from January 1997 to April 2024. Studies were eligible for inclusion if they reported cases of urethral diverticulum (UD) diagnosed during pregnancy and provided detailed clinical, diagnostic, therapeutic, and outcome data. Articles were excluded if they lacked sufficient clinical detail, were published in languages other than English, or involved non-human subjects.

**Figure 6:**
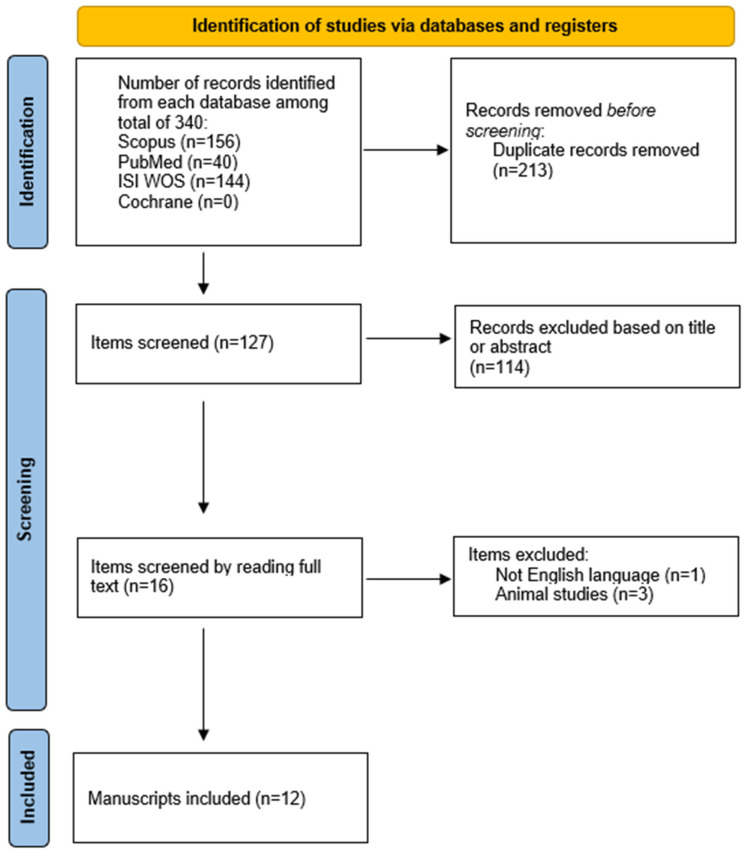
PRISMA 2020 flow diagram for new systematic reviews which include searches of databases and registers only.

Following a thorough screening and eligibility assessment, a total of twelve case reports were identified that met the inclusion criteria and were included in the final synthesis. The PRISMA 2020 checklist for this review is presented in Figure 6.

The identified studies were analyzed and synthesized into a comparative table, focusing on clinical presentation, diverticulum characteristics, diagnostic methods, management strategies during pregnancy, mode of delivery, and postpartum interventions.

A detailed comparison of these cases is presented in Table 1 highlighting the variability in clinical presentation, diagnostic timing, management approaches during pregnancy, and postpartum outcomes.

### 4.1. Clinical Presentation

The symptoms reported were highly heterogeneous. Some women were asymptomatic, with UD discovered incidentally during routine examinations (e.g., Iyer et al., Moran et al. Cases 2 and 3, Xie et al.). Others presented with urinary symptoms such as dysuria, urinary frequency (Moran Case 5), pelvic pain (Carswell), or suprapubic pain with purulent discharge (Jeong). Hematuria and dyspareunia were also noted in specific cases (Moran Case 4, Wittich).

### 4.2. Diverticulum Size

The size of the diverticula ranged from 1.2 cm to over 6 cm. Larger diverticula, particularly those above 4–5 cm, were more likely to cause complications or require intervention before or during delivery.

### 4.3. Diagnostic Approach

Most cases were diagnosed using transvaginal ultrasound (TVUS), often supplemented by three-dimensional imaging (Jeong et al.) or MRI (Carswell, Aoun) when additional anatomical detail was needed. Diagnostic timing varied significantly, with some diverticula identified as early as the first trimester (MRI at 11 weeks, Carswell) and others diagnosed close to term (TVUS at 38 + 4 weeks, Magann et al.).

Accurate imaging is crucial for diagnosing and managing urethral diverticulum (UD) during pregnancy to minimize maternal and fetal risks. Ultrasound (US) is often the first-line imaging modality due to its safety, accessibility, and cost-effectiveness. It effectively identifies periurethral cystic lesions without radiation exposure, making it suitable for pregnant patients [14]. However, US is operator-dependent and may have limited sensitivity in detecting complex or deep diverticula. Magnetic resonance imaging (MRI) offers superior soft tissue contrast and multiplanar imaging capabilities, allowing detailed visualization of the diverticular size, extent, and relationship to surrounding structures, without exposing the fetus to ionizing radiation [15]. MRI is especially valuable in complex or unclear cases and can aid in delivery planning by precisely characterizing the diverticulum and associated complications [16]. Given these advantages, MRI is recommended as a complementary modality following ultrasound in pregnant patients with suspected UD to guide clinical decision-making and optimize outcomes.

### 4.4. Management During Pregnancy

UD during pregnancy is typically managed conservatively. Antibiotics are commonly used, with aspiration or drainage reserved for symptomatic cases. Surgical excision is rare and usually delayed until postpartum unless symptoms are severe (e.g., Iyer, Wittich). Given its rarity (0.02–6% prevalence), patients often require thorough counseling on UD and its associated risks and treatment options [1,3]. While diverticulectomy is the standard outside pregnancy (70–97% success) [1,2,3], its risks include fistula (3–5%), stricture, SUI (10–20%), UTIs, and recurrence (2–16%) [3,4,5,7,8]. During pregnancy, conservative management is favored to avoid surgical and anesthetic risks. In a review of 12 cases, over 80% were managed conservatively until postpartum [13], though recurrence may require later surgery.

In the reported case, the patient opted for non-surgical management and vaginal delivery after shared decision-making. She was stable, with no infection, and risks of complications during labor were discussed. A multidisciplinary team ensured individualized, coordinated care throughout.

### 4.5. Mode of Delivery

The presence of a UD influenced the delivery method in some cases. Cesarean section was preferred in cases with a large diverticulum, to minimize the risk of rupture or fistula formation (e.g., Iyer, Moran Cases 2 and 5, Magann). However, vaginal delivery was successfully achieved in many cases, particularly when the diverticulum was small or asymptomatic (e.g., Moran Cases 1 and 4, Artis, Carswell, Jeong).

### 4.6. Postpartum Intervention

Postpartum follow-up is essential to reassess the need for definitive treatment. Patients should be advised that while conservative management may suffice during pregnancy, recurrence or persistent symptoms are not uncommon and may require surgical intervention once the patient has recovered from childbirth.

In most cases, definitive surgical treatment (i.e., diverticulectomy) was delayed until after delivery. No major recurrences were reported following postpartum surgery, highlighting the effectiveness of delayed intervention in ensuring good maternal outcomes.

### 4.7. Future Directions and Clinical Recommendations

Given the rarity and complexity of UD during pregnancy, future research should focus on establishing standardized imaging protocols and delivery planning strategies. Advanced imaging modalities—such as high-resolution pelvic MRI—can improve diagnostic accuracy and help delineate the anatomical relationship between the diverticulum, urethra, and reproductive structures. Prospective studies are needed to evaluate the optimal timing and modality of imaging to support individualized care planning.

Additionally, guidelines for the mode of delivery remain underexplored. While conservative management is often effective, multidisciplinary consultation involving urologists, radiologists, and obstetricians is recommended to assess risks and determine the safest delivery method, especially in cases with large or symptomatic diverticula. Longitudinal studies tracking maternal and neonatal outcomes could inform best practices and refine counseling for affected patients.

## 5. Conclusions

Urethral diverticulum (UD) during pregnancy remains an elusive and rare diagnosis, often overshadowed by common pregnancy-related symptoms [3,4,5]. Its subtle presentation challenges clinicians, but advances in imaging—especially transvaginal and 3D ultrasound, alongside MRI—have revolutionized detection, offering detailed insights crucial for tailored management without risking fetal safety [3,5,10,11,14,15,16].

While conservative treatment dominates during pregnancy, with surgery reserved for complicated cases, postpartum surgical excision generally yields excellent outcomes [5,8,10,11,13]. This case underscores the critical role of precise imaging and collaborative, patient-centered care in navigating the delicate balance between maternal and fetal well-being.

Given the scarcity of cases and lack of standardized guidelines, ongoing research and multidisciplinary dialog are essential to refine diagnostic strategies, optimize delivery planning, and improve long-term outcomes for this rare but impactful condition.

## Figures and Tables

**Table 1:** Summary of reported cases of urethral diverticulum during pregnancy.

Case Reference	Clinical Presentation	Diverticulum Size	Diagnostic Approach (Gestational Week)	Management During Pregnancy	Mode of Delivery (Gestational Week)	Postpartum Intervention
Iyer et al. [6]	Asymptomatic anterior vaginal mass	3–4 cm	TVUS at 30 + 2 weeks	Surgical excision at 31 + 2 weeks (spinal anesthesia)	Cesarean at 39 weeks (to prevent fistula)	No recurrence reported
Moran et al. [7]—Case 1	Urethral discomfort	1.2 cm	TVUS (2nd trimester)	Antibiotics	Vaginal delivery (week not specified)	Diverticulectomy
Moran et al. [7]—Case 2	Asymptomatic	Not available	Clinical suspicion at 30 weeks	Expectant management	Cesarean at 38 weeks	Diverticulectomy
Moran et al. [7]—Case 3	Asymptomatic	3 cm	Not reported	Needle aspiration	Repeat cesarean	Diverticulectomy
Moran et al. [7]—Case 4	Hematuria	6 cm	TVUS at 30 weeks	Conservative; aspiration during labor	Vaginal delivery	Diverticulectomy
Moran et al. [7]—Case 5	Dysuria, urinary frequency	4 cm	Cystourethroscopy at 6 weeks	Antibiotics, incision and drainage	Cesarean at 37 weeks	Continued I&D post-delivery
Artis et al. [8]	Recurrent symptoms over 3 pregnancies	1–2 cm (range)	TVUS (26–33 weeks)	Conservative/lithotripsy/stents	Three vaginal deliveries	Diverticulectomy after 3rd pregnancy
Wittich [9]	Tender swelling, dyspareunia	Not available	Pelvic exam at 19 weeks	Surgical removal of UD calculi	Not documented	No recurrence reported
Carswell [10]	Severe pelvic pain	2 cm	MRI at 11 weeks	Cystoscopic drainage	Vaginal at 37 weeks	Diverticulectomy
Xie et al. [11]	Asymptomatic vaginal mass	2 cm	Aspiration at 14 weeks	Antibiotics and aspiration	Attempted vaginal → cesarean (labor failure)	Diverticulectomy
Magann et al. [12]	Incidental finding	~5 cm	TVUS at 38 + 4 weeks	Conservative observation	Cesarean at 39 weeks	Reconstructive surgery
Jeong et al. [5]	Suprapubic pain, purulent discharge, leakage	5.5 cm	TVUS, TPUS, 3D ultrasound at 34 weeks	Antibiotics; aspiration considered	Planned vaginal delivery	Postpartum diverticulectomy
Aoun et al. [13]	UTI (58%), purulent discharge (50%), bulging/dyspareunia (33%)	Median 24.6 mm (12–34 mm)	MRI (100%), TVUS (rare), VCUG (33%)	Conservative; UDp milking in 2 cases	11 vaginal, 1 emergency C-section (fetal bradycardia)	Diverticulectomy 3 months postpartum

## Data Availability

The data presented in this study are available on request from the corresponding author.
